# A tutorial on automatic post-stratification and weighting in conventional and regression-based norming of psychometric tests

**DOI:** 10.3758/s13428-023-02207-0

**Published:** 2023-08-21

**Authors:** Sebastian Gary, Wolfgang Lenhard, Alexandra Lenhard, David Herzberg

**Affiliations:** 1 Test Development Center, Psychometrica, Dettelbach, Bavaria, Germany; 2 Wolfgang Lenhard, Institute of Psychology, Julius-Maximilians-University of Würzburg, Bavaria, Germany; 3 WPS, Torrance, CA USA

**Keywords:** Raking, Post-stratification, Regression-based norming, Iterative proportional fitting, Test construction

## Abstract

Norm scores are an essential source of information in individual diagnostics. Given the scope of the decisions this information may entail, establishing high-quality, representative norms is of tremendous importance in test construction. Representativeness is difficult to establish, though, especially with limited resources and when multiple stratification variables and their joint probabilities come into play. Sample stratification requires knowing which stratum an individual belongs to prior to data collection, but the required variables for the individual’s classification, such as socio-economic status or demographic characteristics, are often collected within the survey or test data. Therefore, post-stratification techniques, like *iterative proportional fitting* (= raking), aim at simulating representativeness of normative samples and can thus enhance the overall quality of the norm scores. This tutorial describes the application of raking to normative samples, the calculation of weights, the application of these weights in percentile estimation, and the retrieval of continuous, regression-based norm models with the cNORM package on the R platform. We demonstrate this procedure using a large, non-representative dataset of vocabulary development in childhood and adolescence (*N* = 4542), using sex and ethnical background as stratification variables.

Norm scores allow the comparison of a person’s individual test result with an appropriate reference group (Moosbrugger & Kelava, 2012). In psychodiagnostics, norm scores are often used as a criterion for decisions that have far-reaching consequences for the individual being evaluated, such as school placement based on IQ test results or providing remedial services for learning disorders (American Psychiatric Association, 2013). Consequently, the computation and inclusion of high-quality norms is a crucial aspect of high-standard psychological tests, but it is also an ongoing challenge in test construction.

Since the exact distribution of raw scores in the reference population is usually unknown, norm scores cannot be directly computed. Instead, the norm scores must be derived from a normative sample, that is, a much smaller, representative subsample of the reference population. To this end, statistical methods designed for the norm score computation must be applied to the raw scores (Cole, 1988; Gary et al., 2021). In recent years, advanced norming approaches have been developed and evaluated with respect to their influence on the norm score quality (for an overview, see Gary & Lenhard, 2021). In many psychometric tests (e.g., intelligence scales), the norm scores refer only to individuals of the same age. Therefore, conventional approaches usually split the normative sample into several distinct age groups. In contrast, continuous norming approaches, such as the regression-based norming approach implemented in the R package cNORM (Lenhard et al., 2018a, 2018b), model the distribution of the raw scores as a function of explanatory variables (e.g., age and grade level). Simulation studies have demonstrated the superiority of regression-based norming over conventional methods (Gary & Lenhard, 2021; W. Lenhard & Lenhard, 2021; Oosterhuis, 2017; Voncken, et al., 2021). In this tutorial, we will demonstrate how to automatically integrate post-stratification and weighting in the norming process with the cNORM package to reduce the bias caused by non-representative normative samples.

## The problem of non-representativeness

The quality of norm scores heavily depends on the chosen norming method. In contrast to conventional norming of each distinct group in a norm sample, continuous norming refers to statistical techniques like regression that draw on the complete sample to model the norm scores in dependence of explanatory variables like age and thus allow deliberately fine-grained norms. This can considerably increase the quality of the resulting norm scores and facilitate norming projects due to the higher statistical power, as continuous norming requires a much smaller sample size than conventional norming to achieve the same precision (Lenhard & Lenhard, 2021; Oosterhuis, 2017; Zhu & Chen, 2011). Moreover, the method can even smooth out local violations of representativeness (Gary et al., 2023). However, continuous norming cannot compensate for a general lack of representativeness in the normative sample. The logistics of data collection in test construction are complex. In particular, ensuring that the normative sample is representative of the reference population might be difficult. A sample is representative with respect to the relevant stratification variables (SVs) if the proportions of the various subgroups in the sample match the proportions of the respective strata in the reference population (Kruskal & Mosteller, 1979; Moosbrugger & Kelava, 2012). In other words, representativeness is established when the marginal and joint probabilities of a set of relevant SVs equal the corresponding proportions in the reference population. For example, a sample of Canadian subjects 55% English respectively 21% French mother tongue speakers (24% other languages) would be representative, while a sample of 70% English and 10% French would indicate a violation of representativeness clearly (Statistics Canada, 2022).

When representativeness has not been established with respect to variables that correlate with the latent ability to be measured, the non-representative normative samples can reduce the quality of norm scores and therefore the validity of psychological test results (Gary et al., 2023; Lenhard et al., 2018a, b). For example, Hernández et al. (2017) showed that parental educational level is a predictor of children’s cognitive ability. Therefore, they recommend including parental educational level as a SV in the normative samples of cognitive tests for children, like intelligence, language skills, or school aptitude. If representativeness of the normative sample is neglected regarding such variables, the distribution parameters may be distorted in various ways. Consequently, the norm scores may generally be too high or too low, or they may be biased for a specific range of percentiles (Gary et al., 2023). In the following section, we will describe how to correct or at least mitigate the biases of norm scores introduced by non-representative samples.

## Countering non-representativeness in normative samples

### Probability sampling and sample stratification

Probability or random sampling is probably the best-known strategy for establishing representativeness of normative samples. The data are drawn such that every individual in the population has the same chance to be included in the normative sample (Lumley, 2011). For example, when collecting normative data for a reading comprehension test in elementary school in the U.S., a psychometrician would need to theoretically ensure that every single student at every elementary school has the same chance to be included in the normative sample. In most cases, this sampling is unrealistic because it is not only very time-consuming and cost-intensive, but it could also neglect ethical guidelines (i.e., consent of students and parents, gatekeeper approval).

A more parsimonious method is stratification. To apply this method, the reference population must first be divided into homogeneous subgroups, so-called strata. These strata may be based on one or several SVs. In a second step, a specified number of cases is drawn randomly from each of these strata in such a way that the resulting normative sample is representative of the different strata proportions in the reference population. For example, to obtain a sample of 1000 individuals that is representative of the variable sex (51% men / 49% women), the population is split into a male and a female stratum and the required number of cases (510 men and 490 women) is then drawn from each stratum separately (Moosbrugger & Kelava, 2012). When more than one SV is used, perfect representativeness can only be achieved if all combinations of the different levels of SVs are considered. That is, joint probabilities instead of marginal probabilities must be used to match the proportions of the different strata in the reference population.

Sample stratification requires knowing which stratum an individual belongs to prior to data collection, but the required variables for the individual’s classification, such as socio-economic status or demographic characteristics, are often collected within the survey or test data (Lumley, 2011; Mercer et al., 2018). Moreover, there may be too many SVs or cross-combinations of the different levels of the variables. For example, using three variables with four levels each would result in $4\times 4\times 4=64$ different strata, with some of the combinations possibly being extremely rare. Such a high number of strata increases the required sample size dramatically, which is likely to be far beyond the resources available for a specific norming project. In addition, certain combinations of SVs may not be accessible. Finally, when data are collected in clusters, as is often the case with school achievement tests, systematic drop-outs can occur. Therefore, meeting all the requirements necessary to establish representativeness is often impossible with limited resources. Nevertheless, to minimize the bias caused by unstratified samples, so-called post-stratification methods have been proposed (Lumley, 2011).

### Raking – a post-stratification countermeasure for non-representativeness

The term stratification usually refers to the selection of individuals for data collection, whereas the term post-stratification refers to techniques aimed at improving the representativeness after the data collection has finished. One method to address non-representative data involves randomly reducing cases from overrepresented strata until the desired sample composition is attained. To our knowledge, many test developers are hesitant to discard data and instead prioritize increasing the norm sample size, as naïve test users may perceive this as an indicator of quality. Furthermore, authors may be highly reluctant to relinquish data that was challenging to obtain, even if it is unrepresentative and does not contribute to improved norm data. In past norming projects, we opted to prioritize quality over quantity, collecting an excess of 30–50% of cases, which allowed for subsequent sample stratification. The most commonly administered test of school performance in Germany, ELFE-II (Lenhard et al., 2017), is constructed in this manner. However, this approach has notable drawbacks, as a substantial number of cases must be excluded, leading to a significant reduction in the original sample size and, consequently, a loss of statistical power. As a result, this strategy can only be applied to relatively large samples (e.g., Lenhard et al., 2015) and at the cost of sacrificing relevant portions of norm data, necessitating an accompanying increase in resources.

Another approach to enhance representativeness after data collection involves the use of weighting (Gary et al., 2023). Weighting assigns a weight (*w*) to each test result, effectively treating it as if it were obtained by multiple individuals rather than a single participant with an equal weight (i.e., *w* = 1 for each participant). This weight, *w*, can either increase or decrease a participant's contribution (i.e., w is greater or less than 1). To achieve representativeness, the weight *w* should be equal to the ratio between the proportion of a particular stratum in the reference population and its proportion in the collected sample (Lumley, 2011; Mercer et al. 2018).

This procedure can be easily applied with only one SV, but more than one SV would require knowledge of the joint probabilities, that is, the probabilities of all cross-classifications of the SVs. Moreover, each combination would need to be present in the data. Unfortunately, these requirements often are not fulfilled. A method that requires none of these prerequisites is the so-called *raking* approach. The method is also called *iterative proportional fitting* (Lumley, 2011) because the weights in this process are determined iteratively for only one variable in each step. In the following section, we will illustrate this process with an example based on Mercer et al. (2018). Consider the marginal probabilities of the SVs *sex* and *parental education* in a reference population. The population consists of 48.3% men and 51.7% women. The parental education is low in 40% of the cases, medium in 31% and high in 29% of the cases. The joint probabilities in the actual normative sample are indicated in Table 1.

**Table 1:** Joint distributions of a fictitious normative sample

Parental education	Sex		
	Women	Men	$\Sigma$
Low	15.33%	20.00%	35.33%
Medium	19.17%	18.82%	35.00%
High	17.17%	12.50%	29.67%
$\Sigma$	51.67%	48.33%	100%

First, weights are computed for the variable *sex*, that is, the resulting weights are calculated such that the normative sample simulates the population with respect to the proportions of men and women (see Table 2, step 1). Second, the weights are adjusted to match the respective proportions for *parental education* without considering the SV *sex*. (see Table 2, Step 2). After the weights have been adapted for *parental education*, they might not represent the exact sex proportion in the reference population anymore. Therefore, the process restarts and is repeated until the raking weights have converged (see Table 2, step 5 and step 6). As simulations show, the raking weights usually converge within the first 5–20 steps, with the number of necessary iterations increasing with the number and levels of the SVs (Battaglia et al., 2009).

**Table 2:** Iterative adaption of raking weights

Step	Parental education	Sex	
		Women	Men
0	Low	1.0000	1.0000
	Medium	1.0000	1.0000
	High	1.0000	1.0000
1	Low	0.9290	1.0759
	Medium	0.9290	1.0759
	High	0.9290	1.0759
2	Low	1.0391	1.2033
	Medium	0.8266	0.9573
	High	0.9165	1.0613
3	Low	1.0498	1.1921
	Medium	0.8351	0.9483
	High	0.9260	1.0514
4	Low	1.0514	1.1939
	Medium	0.8346	0.9476
	High	0.9247	1.0500
5	Low	1.0516	1.1937
	Medium	0.8347	0.9475
	High	0.9249	1.0499
6	Low	1.0516	1.1938
	Medium	0.8347	0.9475
	High	0.9249	1.0499

Step 0 represents the non-weighted approach (all weights = 1). Weights are then iteratively adjusted until the marginal proportions of the dataset match the composition of the target population

### Limitations and recommendations for the usage of weighting

While weighting has the potential to reduce the adverse effects of non-representative normative samples on norm score quality, we strongly recommend its thoughtful application and limiting its use to cases where random sampling is not feasible, such as due to logistical issues, clustered data collection, or restrictions in accessing all strata.

First and foremost, while post-stratification serves as a corrective method for improving norm sample data, it cannot replace careful planning and execution of data collection. The primary goal of norming studies should be to achieve the highest possible representativeness of the collected normative sample in terms of demographic and other potentially relevant stratification variables. Failing to do so may result in the undersampling of specific strata (e.g., disproportionately low numbers of non-native speakers for norming vocabulary tests), which, when combined with weighting, may lead to increased norm score errors. This issue arises because raking weights are proportional to the ratio between expected and actual proportions in the normative sample, so strong underrepresentation results in high raking weights.

Test developers should also avoid using stratification variables with many levels, some of which may contain very low expected probabilities. Low probabilities may result from considering stratification variables with many levels or using dummy variables that represent combinations of two or more stratification variables (e.g., combining sex and migration background). In these cases, even well-collected normative samples may contain only a small absolute number of subjects in certain categories, again leading to high weights.

As a practical solution, we recommend combining different levels of a variable if the actual proportions in the normative sample are low and the levels are not expected to differ significantly in mean location. Furthermore, when using dummy coding to incorporate cross-combinations of stratification variables, the number and levels of combined variables should not be too high. This is because the number of dummy-coded levels increases rapidly due to "combinatorial explosion" (Bellman, 1957), which can result in the aforementioned low level probabilities. Based on our experience, weighting works well for instance when using three stratification variables, each with 2 to 3 levels, as long as individual strata comprise at least 5% of the population. However, this observation is based on unsystematic experience.

Finally, the raking approach relies solely on marginal probabilities—namely, the proportions of each SV level within the population. As such, precise knowledge of the SVs' joint probabilities in the reference population is not utilized. However, our simulation study (Gary et al., 2023) indicated that raking in combination with continuous norming should mitigate the adverse effects of non-representativeness, even if only the marginals and not the joint probabilities are applied. This is presumably due to the influence of joint probabilities on the marginals, which are accounted for during the raking process. In case there is reason to belief, joint probabilities have to be represented, it is possible to recode the combinations of the SVs into different strata and use these combined SV in the raking process.

### Combining raking with regression-based or conventional norming

In this tutorial, we demonstrate the utilization of weights in the cNORM package in R. Contrasting traditional discrete norming methods that form norm groups based on discrete age intervals, continuous norming estimates norm scores across a continuous variable like age. The primary concept behind cNORM is fitting a model that captures the relationship between raw test scores and a continuous variable, such as age, using a regression-based approach based on Taylor polynomials. For this, powers of the explanatory variable (e.g., age) and powers of location as well as all linear combinations of these variables are used to predict the raw score, essentially approximating a three-dimensional hyperplane to the data. The procedure so far consisted of (a) determining percentiles of the raw score distribution and (b) transforming them into preliminary manifest norm scores by means of inverse normal transformation (INT), just as it is the case in conventional norming to determine subsample based normal scores. The statistical modelling of cNORM then (c) uses polynomial regression to continuously model raw scores as a function of the norm scores and age or other explanatory variables. Once this norm score model is established, arbitrarily fine-grained norms can be determined. The method reduces the necessity for large norm samples, as it relies on the complete sample rather than distinct groups. It only requires continuity of the dependent variable and does not make distribution assumptions. Thus, it can effectively handle skewed distributions, which frequently arise in test construction due to ceiling and floor effects (Lenhard et al., 2019). The cNORM package has recently been extended to be able to weight the cases. The weights are used in steps (a) and (c) of the norming process. Furthermore, a new function has been added that can be used to generate the required weights for a specific normative sample by means of raking.

In the following section, we explain the integration of weighting in the cNORM package in the following three steps:

1. Computation and standardization of weights via raking.
2. Weighted ranking of the test raw scores using the standardized raking weights
3. Weighted multiple regression to generate a norm model. (optional)

We then demonstrate the whole process using a real data set.

#### Step 1: Computation and standardization of weights via raking

To generate weights with the raking approach, the function ‘computeWeights()’ in the cNORM package can be used, which determines the raking weights and standardizes them to values equal to or larger than 1. For this purpose, the marginal probabilities of all SVs (e.g., sex, parental education, region, or ethnicity) in the reference population must be known. The weights can then be interpreted as how many times the according case counts in the norming process.

#### Step 2: Ranking of the test raw scores using the standardized raking weights

To apply the generated weights, they must be passed via the parameter ‘weights’ in the ‘cnorm()’ function. This function ranks the data groupwise and converts the ranks into percentiles. It subsequently applies inverse normal transformation (INT) to convert percentiles to preliminary norm scores. Finally, multiple regression is performed to establish a continuous norm model. Hence, the ‘cnorm()’ function performs step 2 and step 3 in one single process. If the data are to be analyzed with conventional norming only (i.e., rank-based INT per age group) or if step 2 and step 3 are to be performed separately, the ‘rankByGroups()’ function can be used instead. If a ‘weights’ vector is passed to one of both functions, the ‘weighted.rank()’ function of the cNORM package will be used to determine the ranks of the different raw scores instead of usual ranking. This function counts how often each raw score occurred, taking into account the weights. That is, instead of the actual number of cases in the normative sample, the respective weights are summed. In the case of ties, average ranks are assigned to the corresponding raw scores. Subsequently, the weighted ranks are converted into percentiles. To this end, several standard methods such as Blom, Tukey, Van der Warden, Rankit and others have also been implemented in the cNorm package (parameter ‘method’). Finally, manifest norm scores are computed with INT for every case in the normative sample.

Both the ‘cnorm()’ function and the ‘rankByGroups()’ function return the original observed data, weighted percentiles, manifest norm scores, and the weights for every case in the normative sample.

#### Step 3 (optional): Regression-based norming with inclusion of the raking weights

Finally, the standardized raking weights are applied in a multiple regression to estimate a continuous norm model. More specifically, the regression-based norming approach implemented in the ‘cnorm()’ function models the raw scores as a function of the preliminary norm scores and the explanatory resp. grouping variable. The approach is based on the principle of the so-called Taylor polynomials (cf. Dienes, 1957). In short, this principle states that any smooth function can be approximated by a polynomial. Therefore, the raw scores are regressed on powers of the manifest norm scores up to a power parameter *k*, powers of the explanatory or grouping variable up to a power parameter *t*, and all interactions between powers of the manifest norm scores and explanatory variable. The approach and its related benefits are described in more detail in A. Lenhard et al. (2018a, b, 2019), Lenhard & Lenhard (2021) and Gary et al. (2021). To be able to balance model fit and parsimony, the ‘cnorm()’ function draws on best-subset regression (package *leaps*; Lumley & Lumley, 2013). The best-subset regression returns a sequence of regression models with an ascending number of terms. Each regression model represents the best selection of predictors for a given number of terms in the regression function. Choosing a model with a higher number of terms increases *R*^2^ but also carries the risk of overfitting. To prevent the latter, cNORM offers percentile plots for visual inspection, fit indices like *R*^*2*^, *Malow’s Cp* and *BIC*, and procedures for repeated cross validation. By default, the first model exceeding *R*^2^ > .99 is selected. In most cases, regression models with 4–5 terms already meet this criterion, leading to parsimonious yet well-fitting statistical models.

Researchers disagree about whether stratification weights should be used as weights in regression analyses (DuMouchel & Duncan, 1983; Skinner & Mason, 2012). We nevertheless consider the application of raking weights in the multiple regression to be useful because the regression otherwise risks overfitting the data in areas of high data density and underfitting the data in areas of low data density. We were able to demonstrate in simulation studies that using the weights in the regression improves the norm quality under most conditions (Gary et al., 2023), with only a small risk of introducing new biases.

The ‘cnorm()’ function automatically applies weights in the regression analysis in case they are available. Finally, the resulting continuous norming models can be used to generate norm score tables or to directly transform raw scores to norm scores or vice versa for individual cases with the desired precision.

## Step-by-step example: Weighted norming of a vocabulary test

### Normative sample and reference population

In this section, we illustrate the introduced weighted norming process in a step-by-step example based on a non-representative normative sample of the Peabody Picture Vocabulary Test (PPVT-IV, German adaption; A. Lenhard et al., 2015). The example dataset is already included in the cNORM package and can directly be retrieved using the statistical software R. All code is available in the appendix of the paper.

In our example, we first load the package and assign the PPVT-IV data to the object ‘data’ (cf. Appendix). The data set contains *N* = 4542 cases with raw scores on receptive vocabulary knowledge (ranging from 0 to 228), spanning an age range from 2.6 years to 17.0 years. It includes the variables *sex* (1 = male, 2 = female), *migration* (i.e., migration to Germany from another country; 0 = native, 1 = non-native), and *region* (west, south, north, east), followed by the variable *raw*, which contains the raw score of the test scale. Finally, it includes a grouping variable *group* specifying 15 age groups, which segments the continuous age variable into subsamples spanning 11 months each. The ideal number of groups in a dataset depends on the sample size and also on the functional relation between the dependent variable *raw* and the explanatory variable *age*. During childhood and adolescence, age groups of 6 to 12 months will usually deliver good results for cognitive variables or school achievement. For adults, larger age intervals are appropriate. However, the sample size usually should not be less than 100 per age group.

To calculate weights, we need to generate an additional data frame containing the names of all levels of the stratification variables and the population shares of these levels (i.e., the marginal probabilities) in the reference population. In our example, we use the SVs *sex* and *migration* (cf. Appendix, Step 1a), with a sex ratio of 51 vs. 49% (male vs. female) and migration ratio of 70 vs. 30% (native vs. non-native), which are assigned to the data frame object ‘marginals’. In the data frame, the proportions must be specified for each level of each variable in terms of decimal fractions.

In the given example, the marginal probabilities of the SV *sex* barely deviate from the reference population. Please note that all relevant SVs should nevertheless always be included in the generation of the weights because raking can change the proportions of the individual SVs.

### Computation of raking weights

The weights can subsequently be computed using the ‘computeWeights()’ function by passing the sample data and the population marginals for the SVs as function parameters. The resulting vector ‘weights’ contains a weight for each individual case in the normative sample obtained by raking and subsequent standardization of the weights (Appendix, Step 1b).

#### Ranking with standardized raking weights

In our example, both the ranking and the best-subset regression are conducted with the ‘cnorm()’-function. This function returns an object containing the original data, the weights, the group-specific ranks, the preliminary norm scores, and powers of the norm scores, of the grouping variable and all interactions between them (cf. Gary et al., 2021). The object also contains the final statistical model describing the functional relation between raw scores, norm scores, and the explanatory variable, which is age in the example.

The norm scores are returned as *T* scores (M = 50, SD = 10) by default, but other types of scales such as *z* scores or IQ scores can also be used. By default, cNORM calculates unweighted percentiles but automatically switches to weighted percentiles, if a vector with weights is provided (see description above and Appendix, Step 2). Given that the distribution of the raw scores in the reference population is approximated with this procedure, weighting usually increases the reliability and therefore also the predictive validity of the norm scores (Gary et al., 2023).

#### Regression-based norming with inclusion of the raking weights

**Fig. 1:**
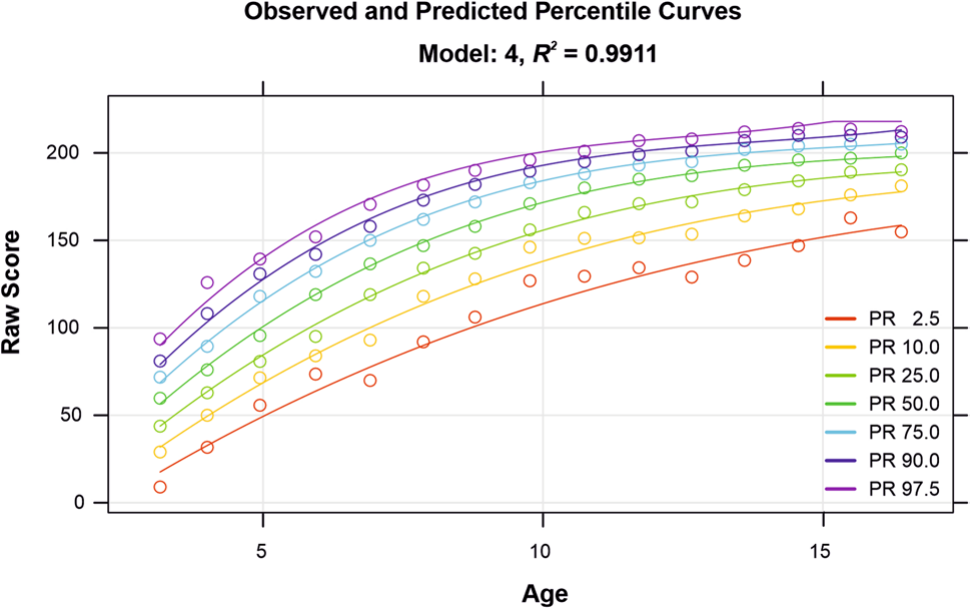
Continuous norm model based on weighted cases of the PPVT-IV dataset. *Note*. The plot depicts the manifest percentiles of the 15 distinct age groups (*dots*) and the fitted percentile curves (*lines*) for a selected set of percentiles ranging from PR 2.5 to PR 97.5. The curves are smooth and do not intersect, which is a requirement for a valid norm model

Finally, the standardized raking weights are automatically used as regression weights to compute the final norm model via multiple regression. The ‘cnorm()’ function performs the regression and the model selection and also provides a graphical illustration of the model in the form of a percentile plot (Fig. 1). In our specific example, the selected function includes the following four terms: *raw* ~ *a* + *la* + *l*^2^*a*^2^ + *l*^3^*a*^3^, with *l* representing the person location (resp. the manifest norm score) in the form of *T* scores and *a* representing age. The adjusted *R*^2^ amounts to .9911. Furthermore, visual inspection of the graphical model illustration shows no inconsistencies such as, for example, intersecting percentile curves, and no signs of overfitting such as wavy percentile curves. As can be seen in Fig. 1, the modelled scores (lines) match the manifest norm scores (dots) very well. To be able to visually inspect and compare different models, we recommend plotting a whole series of models with an ascending number of terms with the ‘plotPercentileSeries()’ function (Appendix, Steps 2 and 3). Among this series of models, for example Model 2, which only includes two terms in the regression function, displays intersecting percentile curves and thus demonstrates, how an inconsistent model could look like. In our specific example, the returned model stands up to scrutiny and the integrated numerical check yields *“No violations of model consistency found.”* If, however, the graphical inspection indicates that another model might even fit better or if the model fit is not satisfying, the ‘cnorm()’ function should be rerun with a fixed number of terms (parameter ‘*terms*’) or different power parameters *k* or *t*, with k specifying the power parameter for the location and *t* relating to the power parameter for the explanatory variable, e. g. age. In our experience, good models are usually obtained by choosing *k* = 5 and *t* = 3. However, the power parameters should be reduced in case of overfit of the data. For example, if the correlation between the explanatory variable and the measured variable is low, *t* = 2 or even *t* = 1 may yield better results.

In summary, the resulting weighted regression model seems to account for a high amount of variance in the normative sample with a high fit and no indications of inconsistencies. The model is now ready to be used for either dynamically retrieving norm scores for individual results (e.g., in computer-based testing), computing norm scores for a complete data set, or generating norm tables for manually scoring test results (cf. Appendix).

## Conclusion

Ensuring the representativeness of normative data is of paramount importance in test construction to avoid biased decisions. Nevertheless, the process can be complex, and obtaining fully representative normative samples is unfeasible. In this case, post-stratification strategies such as raking can help improve the quality of the norms. In this tutorial, we demonstrated the computation and application of raking weights with the cNORM package on the R platform. This package is specifically designed for continuous norming. As demonstrated in simulation studies (Gary et al., 2023), the combination of raking and continuous norming as implemented in the cNORM package in most cases reduces the error introduced in norm scores through non-representative normative samples. Nevertheless, we would like to emphasize that a reduction is not always the case. For example, weighting cannot fully compensate for very high deviations from representativeness. Moreover, the use of weights may even have negative effects if subgroups at the upper and lower tails of the performance range are severely underrepresented. Therefore, weighting should not supplant the effort to acquire a representative sample of sufficient size. We recommend using the method only when deviations from representativeness are moderate at most, if outlying groups are sufficiently represented, and when the sample size is at least 100 per age group.

Finally, it is crucial to remember the broad array of diagnostic approaches at our disposal, with this tutorial focusing primarily on enhancing norm-referenced tests. Such tests are invaluable where no established criteria exist, permitting assessment relative to the norm group. Conversely, when definitive criteria exist, as in obtaining a driving license, measuring individual compliance to these criteria becomes more appropriate. Thus, whilst norm-referenced tests hold a pivotal role in certain contexts, the development of criterion-based tests is an equally imperative endeavor.

## Appendix

The following code demonstrates the step-by-step procedure of the tutorial, using the R platform (R Core, 2022).

## References

[CR1] American Psychiatric Association. (2013). *Diagnostic and statistical manual of mental disorders: DSM-5*. American Psychiatric Association.

[CR2] Battaglia, M. P., Hoaglin, D. C., & Frankel, M. R. (2009). Practical considerations in raking survey data. *Survey Practice,**2*(5), 2953. 10.29115/SP-2009-0019 10.29115/SP-2009-0019

[CR3] Bellman, R. (1957). *Dynamic programming*. Princeton University Press.

[CR4] Cole, T. J. (1988). Fitting smoothed centile curves to reference data. *Journal of the Royal Statistical Society: Series A (Statistics in Society),**151*(3), 385–406. 10.2307/2982992

[CR5] Dienes, P. (1957). *The Taylor series: An introduction to the theory of functions of a complex variable*. Dover Publications.

[CR6] DuMouchel, W. H., & Duncan, G. J. (1983). Using sample survey weights in multiple regression analyses of stratified samples. *Journal of the American Statistical Association,**78*(383), 535–543. 10.1080/01621459.1983.10478006 10.1080/01621459.1983.10478006

[CR7] Gary, S., & Lenhard, W. (2021). In norming we trust: Verfahren zur statistischen Modellierung kontinuierlicher Testnormen auf dem Prüfstand [In norming we trust: Methods for statistical modeling of continuous testing standards on the test bench]. *Diagnostica,**67*(2), 75–86. 10.1026/0012-1924/a000263 10.1026/0012-1924/a000263

[CR8] Gary, S., Lenhard, W., & Lenhard, A. (2021). Modelling Norm Scores with the cNORM Package in R. *Psych,**3*(3), 501–521. 10.3390/psych3030033 10.3390/psych3030033

[CR9] Gary, S., Lenhard, A., Lenhard, W., & Herzberg, D. S. (2023).Reducing the Bias of Norm Scores in Non-Representative Samples: Weighting as an Adjunct to Continuous Norming Methods. *Assessment, Online First.*10.1177/10731911231153832 10.1177/10731911231153832 PMC10623617 36794743

[CR10] Hernández, A., Aguilar, C., Paradell, È., Muñoz, M. R., Vannier, L.-C., & Vallar, F. (2017). The effect of demographic variables on the assessment of cognitive ability. *Psicothema,**29*(4), 469–474. 10.7334/psicothema2017.33 29048305 10.7334/psicothema2017.33

[CR11] Kruskal, W., & Mosteller, F. (1979). Representative sampling, III: The current statistical literature. *Revue Internationale de Statistique, 47*(3), 245. 10.2307/1402647.

[CR12] Lenhard, W., & Lenhard, A. (2021). Improvement of Norm Score Quality via Regression-Based Continuous Norming. *Educational and Psychological Measurement,**81*(2), 229–261. 10.1177/0013164420928457 37929259 10.1177/0013164420928457 PMC10621686

[CR13] Lenhard, A., Lenhard, W., Segerer, R., & Suggate, S. (2015). *Peabody Picture Vocabulary Test (PPVT-4)*. Pearson Clinical Assessment.

[CR14] Lenhard, W., Lenhard, A., & Schneider, W. (2017). *ELFE II-Ein Leseverständnistest für Erst-bis Siebtklässler* [ELFE II - A Reading Comprehension Test for First to Seventh Graders]. Hogrefe.

[CR15] Lenhard, A., Lenhard, W., & Gary, S. (2018a). *Continuous Norming (cNORM)*. The Comprehensive R Archive Network. Retrieved from https://CRAN.R-project.org/package=cNORM. Accessed 10 June 2023.

[CR16] Lenhard, A., Lenhard, W., Suggate, S., & Segerer, R. (2018b). A continuous solution to the norming problem. *Assessment,**25*(1), 112–125. 10.1177/1073191116656437. Accessed 10 June 2023. 27371826 10.1177/1073191116656437

[CR17] Lenhard, A., Lenhard, W., & Gary, S. (2019). Continuous norming of psychometric tests: A simulation study of parametric and semi-parametric approaches. *PloS One,**14*(9), e0222279. 10.1371/journal.pone.0222279 31527877 10.1371/journal.pone.0222279 PMC6748442

[CR18] Lumley, T. (2011). *Complex surveys: A guide to analysis using R* (565th ed.). John Wiley & Sons.

[CR19] Lumley, T., & Lumley, M. T. (2013). Package ‘leaps’: Regression subset selection. The Comprehensive R Archive Network. Available online: http://CRAN.R-project.org/package=leaps. Accessed 10 Aug 2023.

[CR20] Mercer, A., Lau, A., & Kennedy, C. (2018). *For weighting online opt-in samples, what matters most?* Pew Research Center.

[CR21] Moosbrugger, H., & Kelava, A. (2012). *Testtheorie und Fragebogenkonstruktion* [Test theory and questionnaire construction]. Springer.

[CR22] Oosterhuis, H. E. M. (2017). Regression-based norming for psychological tests and questionnaires. PhD thesis, Tilburg University. Available online: https://research.tilburguniversity.edu/files/16257245/Oosterhuis_Regression_12_04_2017.pdf. Accessed 10 Aug 2023.

[CR23] R Core (2022). R: A *language and environment for statistical computing*. R Foundation for Statistical Computing, Vienna, Austria. Available online: https://www.R-project.org/. Accessed 10 Aug 2023.

[CR24] Skinner, C., & Mason, B. (2012). Weighting in the regression analysis of survey data with a cross-national application. *Canadian Journal of Statistics,**40*(4), 697–711. 10.2307/41724556 10.2307/41724556

[CR25] Statistics Canada (2022). While English and French are still the main languages spoken in Canada, the country’s linguistic diversity continues to grow. Available online: https://www150.statcan.gc.ca/n1/daily-quotidien/220817/dq220817a-eng.htm. Accessed 10 June 2023.

[CR26] Velez, J. I., & Correa, J. C. (2014). Should we think of a different median estimator. *Comunicaciones en Estadistica,**7*(1), 11–17. 10.15332/s2027-3355.2014.0001.01

[CR27] Voncken, L., Albers, C. J., & Timmerman, M. E. (2021). Bias-variance trade-off in continuous test norming. *Assessment,**28*(8), 1932–1948. 10.1177/1073191120939155 32659111 10.1177/1073191120939155 PMC8543664

[CR28] Wei, L., Wang, D., & Hutson, A. D. (2015). An investigation of quantile function estimators relative to quantile confidence interval coverage. *Communications in Statistics - Theory and Methods,**44*(10), 2107–2135. 10.1080/03610926.2013.775304 26924881 10.1080/03610926.2013.775304 PMC4768491

[CR29] Zhu, J., & Chen, H.-Y. (2011). Utility of inferential norming with smaller sample sizes. *Journal of Psychoeducational Assessment,**29*, 570–580. 10.1177/0734282910396323 10.1177/0734282910396323

